# On the Dependence of Prion and Amyloid Structure on the Folding Environment

**DOI:** 10.3390/ijms222413494

**Published:** 2021-12-16

**Authors:** Irena Roterman, Katarzyna Stapor, Krzysztof Gądek, Tomasz Gubała, Piotr Nowakowski, Piotr Fabian, Leszek Konieczny

**Affiliations:** 1 Department of Bioinformatics and Telemedicine, Jagiellonian University Medical College, 31-034 Kopernika 7, 30-688 Krakow, Poland; 2 Department of Applied Informatics, Silesian University of Technology, Akademicka 16, 44-100 Gliwice, Poland; katarzyna.stapor@polsl.pl; 3 Sano Centre for Computation Medicine, Czarnowiejska 36, 30-054 Kraków, Poland; krzysztof.gadek15@gmail.com (K.G.); t.gubala@sanoscience.org (T.G.); p.nowakowski@sanoscience.org (P.N.); 4 Department of Algorithmics and Software, Silesian University of Technology, Akademicka 16, 44-100 Gliwice, Poland; Piotr.fabian@polsl.pl; 5 Department of Medical Biochemistry, Jagiellonian University Medical College, 31-034 Kopernika 7, 31-034 Krakow, Poland; mbkoniec@cyf-kr.edu.pl

**Keywords:** amyloids, prions, hydrophobicity, fuzzy oil drop model

## Abstract

Currently available analyses of amyloid proteins reveal the necessity of the existence of radical structural changes in amyloid transformation processes. The analysis carried out in this paper based on the model called fuzzy oil drop (FOD) and its modified form (FOD-M) allows quantifying the role of the environment, particularly including the aquatic environment. The starting point and basis for the present presentation is the statement about the presence of two fundamentally different methods of organizing polypeptides into ordered conformations—globular proteins and amyloids. The present study shows the source of the differences between these two paths resulting from the specificity of the external force field coming from the environment, including the aquatic and hydrophobic one. The water environment expressed in the fuzzy oil drop model using the 3D Gauss function directs the folding process towards the construction of a micelle-like system with a hydrophobic core in the central part and the exposure of polarity on the surface. The hydrophobicity distribution of membrane proteins has the opposite characteristic: Exposure of hydrophobicity at the surface of the membrane protein with an often polar center (as in the case of ion channels) is expected. The structure of most proteins is influenced by a more or less modified force field generated by water through the appropriate presence of a non-polar (membrane-like) environment. The determination of the proportion of a factor different from polar water enables the assessment of the protein status by indicating factors favoring the structure it represents.

## 1. Introduction

The protein folding problem has been the subject of analysis for many years [1,2]. The proposed numerous paths representing the folding process take into account energetic conditions and include entropy phenomena [3]. Amyloids contradict the commonly accepted certainty about structure determination by amino acid sequence. Amyloids are formed without the need for mutation and represent a significantly different structural form resulting in the formation of linear fibrils that do not have much in common with the phenomenon of protein complexation [4,5].

Side chains are mainly involved in protein complexation, while in the structure of amyloids a fundamental role is played by interactions resulting from the abundant presence of beta-structural secondary structures. This structural form is based on a network of interchain hydrogen bonds formed by backbone atoms [6].

It is impossible to mention here the literature on the phenomenon of amyloidosis due to their large number [7]. However, these works will be cited, which constitute the basis for the presented considerations. The basis is the finding of a fundamental structural difference between the globular and amyloid proteins. The most important word here is “fundamental.” In the current analysis, it means a different mechanism of the process of generating the structures of the mentioned proteins [8].

The second important observation concerns the environment and, in particular, the role of water in the folding and complexation processes [9]. The topic of the structural characteristics of water, discussed in [10], discusses the structuring of water by treating individual water molecules.

The FOD model is based on the results of the presence of water, which is expressed in the structure of proteins. The variability of water properties due to the presence of other molecules influences the folding process as bi-polar amino acid molecules are sensitive to the specificity of the surrounding field. A perfect arrangement of the spherical micelle type is possible under the fulfillment of two conditions: the bipolar structure of the dissolved molecule and the properties of water. A polypeptide chain composed of diverse bipolar amino acid molecules with limited mobility (covalent peptide bonds) allows the construction of micelles only under certain conditions. Identification of proteins with hydrophobic distribution consistent with the arrangement present in the spherical micelle proves the correctness of this assumption [11,12]. Additionally, the recently introduced modification of this model (namely the FOD-M model [13]) takes into account the degree of participation of factors other than water influencing the change of the form of the external force field, including the environment of the hydrophobic membrane in particular. The role of external factors is discussed in detail in papers [14,15]. In particular, the role of hydrophobic factors in amyloid transformation with respect to Alpha-synuclein is discussed in [16,17,18,19]. 

The role of the environmental factor determined on the basis of the application of the FOD model has been demonstrated by different characteristics of the native structure of this protein and its amyloid form [13]. With the help of this analysis, it was possible to propose different characteristics for amyloids resulting from the transformation of transthyretin or the V domain of the IgG light chain [13]. The reasoning proposed in [13] is continued in the present work in relation to the prion proteins in their native form and in the amyloid form. The list of analyzed proteins was obtained using the keyword prions in the PDB database [20]. Prion proteins are treated as precursors of numerous amyloids [21,22,23,24].

The present work discusses the characterization of prion proteins in relation to the analogous one based on the analysis of hydrophobicity distribution present in amyloids resulting from prion transformation. The results of this study are a continuation of the study of amyloid structures with a different amino acid composition [13]. 

The subject of the analysis includes prion proteins with single-chain structure [25,26,27,28,29,30,31], those that are partially unfolded [32,33,34]; prion proteins in dimer form [35]; prion-like proteins [36,37]; those in the form of complexes with the Fab IgG fragment [38,39,40]; mono-fibrillary amyloids [41,42,43,44,45,46,47]; and super-fibrillary amyloids [48,49,50,51,52]. Additionally, a representative of proteins in the native form belonging to the hnrnpa family was analyzed in order to enable comparative analysis [53]. Table S1 provides a summary of the analyzed proteins.

## 2. Results

### 2.1. Description of the Model Used—The Fuzzy Oil Drop Model

The fuzzy oil drop model has already been described in detail [12]. Only the information necessary for the interpretation of the results presented here is provided here.

This description is included in the Results section, as without specifying the meaning of the parameters derived from it, it is not possible to interpret the results presented.

The main idea of the model is to treat the protein molecule as a form obtained by bipolar molecules. Such molecules form structures referred to as micelles. Amino acids—molecules with a distinct bipolar structure with a differentiated polarity/hydrophobicity ratio—limited by covalent bonds can obtain such a structure only to a limited extent. It is assumed, however, that despite this limitation, they tend in aqueous media to concentrate hydrophobic residues in the center of the molecule with simultaneous exposure of polar residues on the surface. Such a distribution of hydrophobicity is expressed by a 3D Gaussian function stretched over the protein body with appropriate values of SigmaX, SigmaY and SigmaZ parameters adapted to the size and shape of the protein molecule. The positions of so-called effective atoms (averaged position of the atoms constituting the amino acid) are assigned a value of the 3D Gaussian function expressing an idealized level of hydrophobicity assuming micelle-like distribution of hydrophobicity. The hydrophobicity level thus determined is referred to as Ti.

In fact, the distribution of hydrophobicity does not necessarily reflect such a pattern. The distribution that occurs in protein is the result of inter-amino acid interactions depending on the distance between the effective atoms and on the intrinsic hydrophobicity of each amino acid. The level of hydrophobicity resulting from the actual distribution of high and low hydrophobic residues is referred to as Oi. The function proposed by M. Levitt [54] was used here. The status of Oi distribution is assessed by comparing T and O distributions.

Another reference distribution is uniform distribution in which each residue is assigned the same level of hydrophobicity (Ri = 1/N, where N is the number of amino acids in the protein). This type of decomposition lacking a hydrophobic core is referred to as Ri.

The degree of similarity of the O distribution can be quantified using divergence entropy introduced by Kullback–Leibler—D_KL_ [55]. The D_KL_ value determined for the O|T relation is obtained by assessing the status of the O distribution with reference distribution T. Similarly, the D_KL_ evaluation for the O|R relation determines the degree of similarity of the O distribution with the R distribution treated as the reference distribution.

A higher D_KL_ value for the O|R relation than for the O|T relation means the presence of a hydrophobic core, representing a micelle-like system. In order to eliminate the use of two values for the description of one object, the RD—Relative Distance parameter—was introduced and is expressed as follows.

(1) $$\mathrm{RD}=\frac{D_{\mathrm{KL}}\left(O|T\right)}{D_{\mathrm{KL}}\left(O|T\right)+D_{\mathrm{KL}}\left(O|R\right)}$$

**Figure 1:**
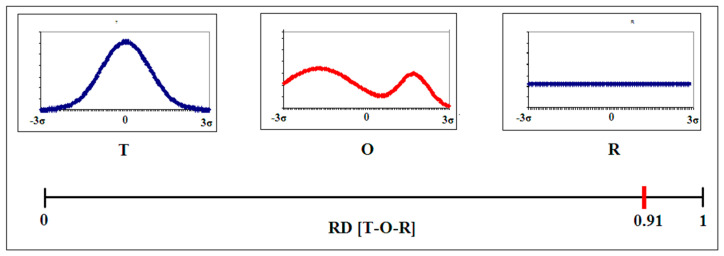
Graphical presentation of determining the value of the RD parameter for the T-O-R relation. The T distribution results from the Gaussian function, the O distribution represents an exemplary observed distribution and the R distribution is a uniform system devoid of variations in the level of hydrophobicity, particularly the central concentration (hydrophobic core). The horizontal axis shows the variability of RD values for the relationship (T-O-R), where the distribution T and R are treated as reference distributions for the observed distribution of O. In the discussed example, this value is 0.91, which means the absence of a hydrophobic core in distribution O. The horizontal axes express spatial coordinates (after normalization it covers the −3Sigma to +3Sigma range). The molecule is localized with its geometric center in the origin of the coordinate system. This explains the values given on the *x*-axis. The same is used in all presentations.

The value RD less than 0.5 indicates the presence of a hydrophobic core. Proteins with a very low RD value have been identified: downhill, fast-folding and antifreeze type III proteins, and the vast majority of domains present in protein structures [11]. This justifies the use of the discussed model for the purpose of protein structure evaluation from the point of view of the influence of the external force field on protein structuring (Figure 1).

**Figure 2:**
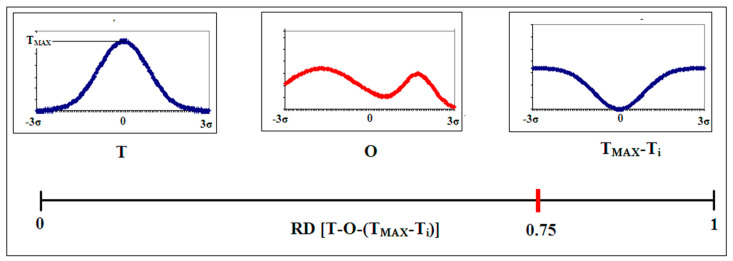
Introduction of a referential distribution of the inverted form of the Gaussian function. T reference distribution—centrally located; observed distribution—O; distribution in the form of T_MAX_-Ti—inverse to the Gaussian distribution. Below is the RD value scale with a mark at the position 0.75, which indicates the status of the O distribution.

**Figure 3:**
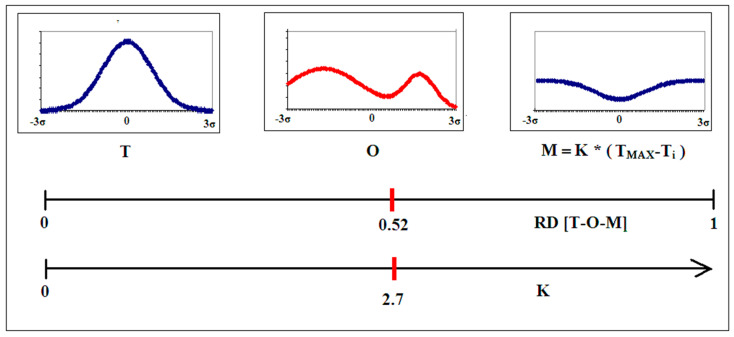
Introduction of the reference distribution M taking into account the degree of involvement of factors other than polar water. Central axis—axis of the RD value for the T–O–M system, where the RD value is given which defines the status of the O distribution against the given reference distributions. The lower axis shows the scale of the K value—K corresponding to the distribution status O is marked. It should be noted that to facilitate the readability of the described model, a simplification in the form of a one-dimensional Gaussian function analysis was used, while the 3D Gaussian distribution is used for the description of proteins.

The formation of micellar structures is specific to the aquatic environment. However, proteins also act in a different environment, which is the hydrophobic environment of the membrane. Here, the distribution is expected to be the inverse of the discussed distribution, based on the 3D Gaussian distribution. In order to describe this situation, a function called M (Modified fuzzy oil drop model) is used (Figure 2 and Figure 3):

(2) $$M_{i}={\left[T_{i}+K\;{\left(T_{\mathrm{MAX}}-T_{i}\right)}_{n}\right]}_{n}$$

where M_i_ is the level of hydrophobicity in the modified field, T_i_ is the idealized level of hydrophobicity, T_MAX_ is the maximum value of T_i_ in the T distribution and index n denotes normalization. The expression T_MAX_-T_i_ represents the inverse distribution observed in membrane-embedded proteins. The K parameter expresses the contribution of this altered external field together with a typical field for the polar environment, providing the distribution M_i_. The distribution of M_i_ is a modified form of the external field with the participation of extra-polar factors. It acts as a target for the folding protein.

Proteins (including domains) showing K = 0 are identified, which means the presence of a structure that is the effect of directing the folding process towards the micelle-like form [12]. These include downhill, fast-folding, ultrafast-folding and antifreeze type III proteins [11]. Such status is also displayed by the vast majority of domains treated as individual structural units (the 3D Gauss function is generated for a domain).

The use of the fuzzy oil drop (FOD) model and its modified FOD-M version visualizes the set of figures in a simplified and reduced form to Gaussian functions (3D Gauss functions are used to describe proteins).

The next set shows the use of the T_MAX_-T_i_ distribution as the reference distribution for the O distribution. The horizontal axis shows the RD value for the T-O-(T_MAX_-T_i_) relationship. The obtained value indicates the “closeness” of the O distribution to the T-O-(T_MAX_-T_i_) distribution.

The principle of the modified version of the FOD model consists in introducing the quantification of the share of the reference distribution T-O-(T_MAX_-T_i_) to the extent defined by parameter K. In the given example for the T–O–M system (the M distribution is the reference distribution instead of the R distribution), the value of RD for T-O-(T_MAX_-T_i_) indicates the proximity of the O distribution to the M distribution (RD = 0.52). The parameter determining the power of the “inverted” field share is expressed with the value of K = 2.7. This means that factors other than polar water contribute significantly to the formation of the O distribution.

The value of K is searched for by identifying the modified distribution with the lowest D_KL_ value for the O-M relation. 

In the discussed example (it should be noted that reduced to a one-dimensional Gaussian function), the value of K introduced a modification of the reference distribution to the form K ∗ [T-O-(T_MAX_-T_i_)]. The value of the K parameter determines the “strength” (degree and power) of the share of the force field different from the force field originating from water (3D Gaussian function). Interpretation of the result obtained is based on the assessment of the characteristics of the environment that induces a structure with the distribution shown as O. In particular, a significant proportion (as in the case of the discussed example) relates to the proportion of the hydrophobic environment of the cell membrane, which is the environment for many proteins functioning as, e.g., ion channels.

It should be noted that the obtained high K value in the discussed example is observed in real proteins for membrane proteins. Globular proteins operating in the water environment are described with a K value close to zero (most often 0 < K < 0.4). Proteins with K = 0 are downhill, fast-folding or antifreeze type III proteins [11]. Most of the domains treated as independent structural units (3D Gauss function generated for the domain) show low K values within the given range.

All values of RD given in the further part of the paper are determined for the T-O-R relation.

### 2.2. Prions

**Figure 4:**
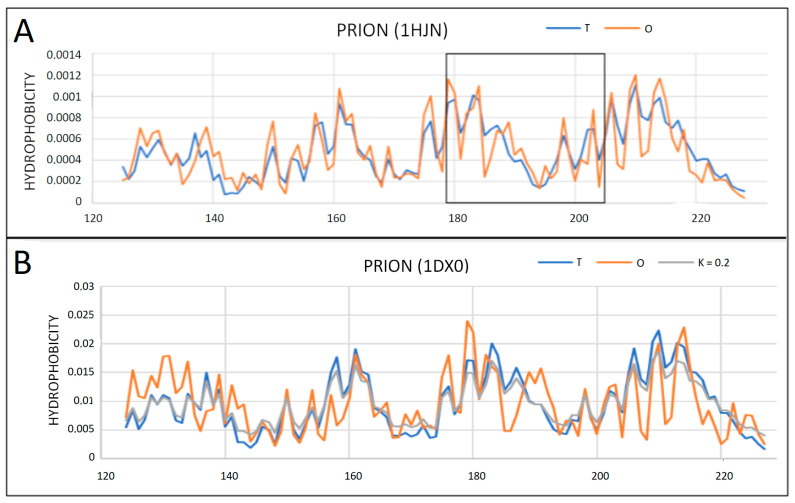
Profiles T, O and M for single-chain prion proteins. (**A**) 1HJN—the section delimited by the disulfide bond is marked with a frame (K = 0 for this distribution); (**B**) 1DX0—the M distribution showing a slight modification of the T distribution is also placed.

The model presented above, applied to single-chain prion proteins, reveals their structure with a well-shaped hydrophobic core, which is expressed by the low values of RD parameter for the T-O-R relation (Table 1). All values of parameter RD below 0.5 and very low values of the K parameter indicate the stabilization of the structure resulting from the presence of the centric, ordered hydrophobic core (Figure 4).

Additionally, tertiary stabilization is also supported by disulfide bonds. The segment connecting the Cys positions that build this bond presents a high degree of adaptation of the hydrophobicity distribution within it to the distribution throughout the entire molecule. This means that both factors responsible for tertiary stabilization are present in the structure of the prion proteins in question.

By observing T, O and M distributions (resulting from K ≠ 0), only slight differences between the T and O distributions can be detected (Figure 4).

**Figure 5:**
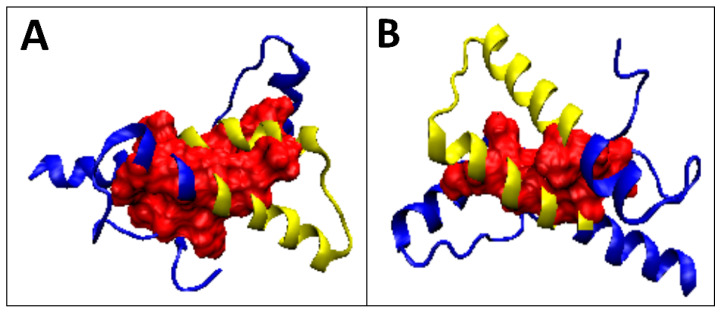
3D presentation of the selected prion proteins: (**A**) 1JN; (**B**) 1DX0. Hydrophobic core marked in red, fragment limited by Cys participating in SS-bond marked in yellow.

The 3D structure very close to the globular one indicates high ordering in accordance with the 3D Gaussian distribution discussed above (Figure 5).

### 2.3. Partially Unfolded Prion Proteins

In the collection of prion proteins, there are examples with a partially unfolded N-terminal fragment (Table 2). From the point of view of the fuzzy oil drop model, the status of these proteins is somewhat disturbed. The disorder comes from a loose part of the chain. The remaining part loses the status of the 3D Gauss distribution with the exception of 5YJ4 and 6FNV, where despite the absence of the N-terminal fragment, the remaining part retains micelle-like distribution. It is worth noting that the segment marked by the Cys building the disulfide bond, in some cases, maintains the decomposition status consistent with the micelle-like distribution (Table 2).

**Figure 6:**
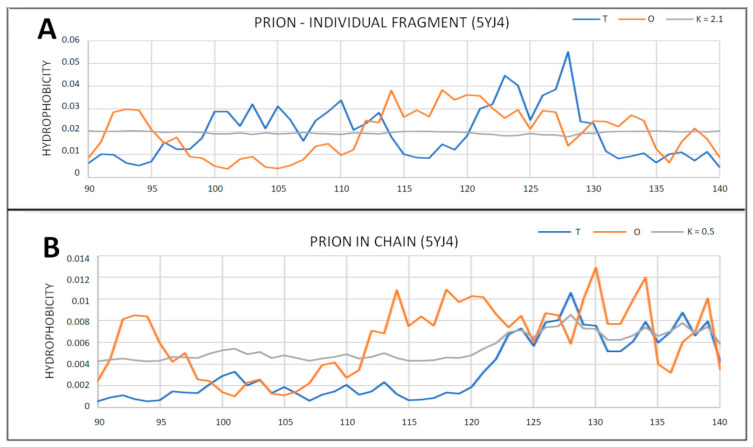
T, O and M profiles for prions with a partially unfolded N-terminal fragment as observed in 5YJ4. (**A**) Complete chain; (**B**) N-terminal segment.

**Figure 7:**
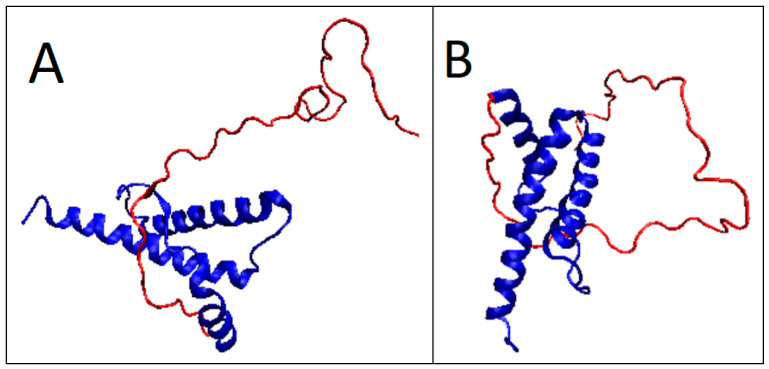
3D presentation of the prion structure with a partially unfolded N-terminal fragment of the chain: (**A**) 5YJ5; (**B**) 5YJ4. The unstructured sections—in red.

In order to show a significant change in the role of the N-terminal fragment in the structure of the entire molecule and of the status of this part of the chain treated as an individual molecule, a summary was provided (Figure 6 and Figure 7). The status of the fragment treated as an individual unit shows an almost linear M distribution. This means—according to the interpretation of the fuzzy oil drop model—obtaining a structure completely independent of the environment. This distribution is not influenced by the centric (3D Gauss) or the inverse (T_MAX_-3DGauss) system.

### 2.4. Prion Protein in Form of Dimers

Prion proteins are also available as homo-dimers (Table 3). Single chains show a status with a hydrophobic centric core (RD < 0.5) and low values of the K parameter (Table 3).

**Figure 8:**
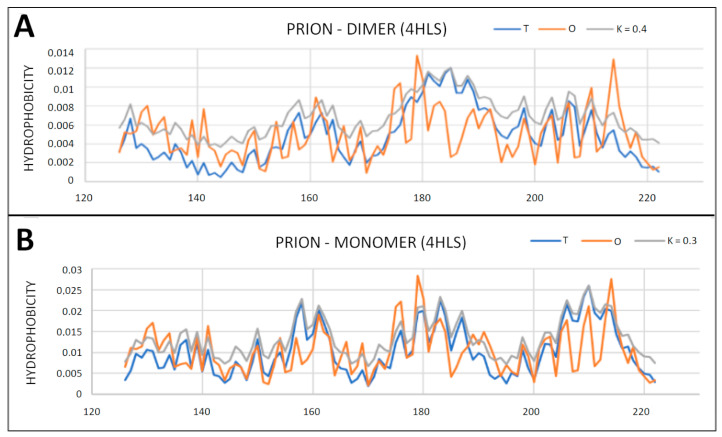
T, O and M profiles for the prion protein for which its structure available in PDB is dimer—4HLS. (**A**) Profiles for the chain A as it shows in dimer; (**B**) profiles for a monomer treated as an individual structural unit.

**Figure 9:**
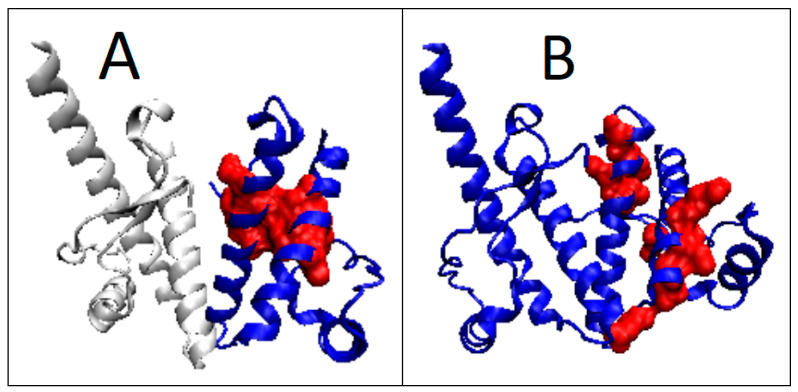
Different hydrophobic core organization in (**A**) monomer and (**B**) dimer as observed in 4HLS.

In contrast, dimers do not show the presence of a common hydrophobic core (Table 3, Figure 8 and Figure 9). Complexation does not take place in these cases by generating a common hydrophobic core. This is due to the presence of a polar surface coating (monomer status). The change of status after switching to the dimer form does not show a significant change in the value of the K parameter, which means that the water environment is sufficiently involved in the formation of the dimers.

### 2.5. Prion-like Proteins

**Figure 10:**
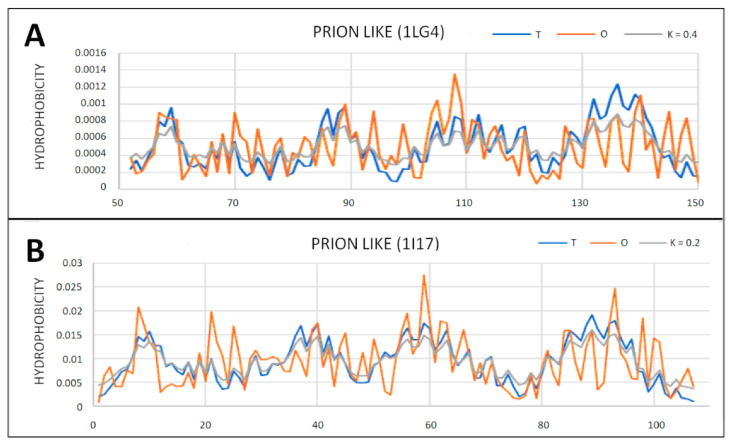
T, O and M profiles for prion-like proteins. (**A**) Protein with the code PDB—1LG4; (**B**) protein with the code PDB—1I17.

**Figure 11:**
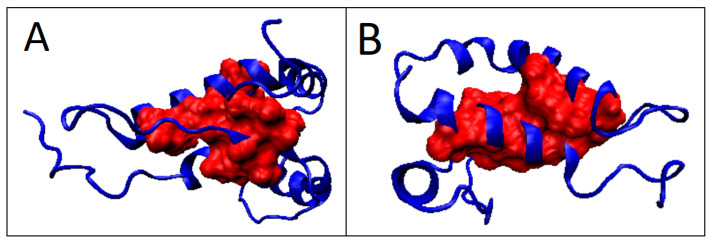
3D presentation of proteins identified as prion-like. (**A**) 1LG4; (**B**) 1I17. Hydrophobic core marked in red.

Prion-like proteins were also analyzed (Table 4). They show a structure in common with prion proteins, with a centric hydrophobic core consistent with the micelle-like system (Figure 10 and Figure 11).

### 2.6. Prion Proteins in Form of Complexes with Fab Fragments of IgG

**Figure 12:**
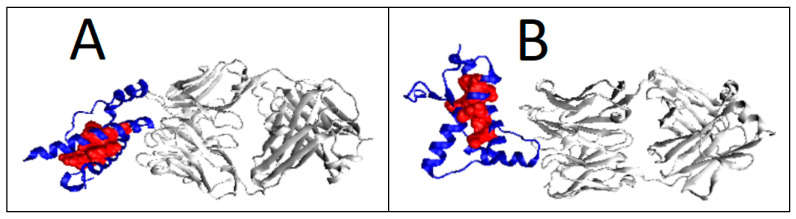
Different organization of the hydrophobic core structure in prion proteins included in the complex with the Fab fragment of IgG. (**A**) 6AQ7; (**B**) 4MA8.

**Figure 13:**
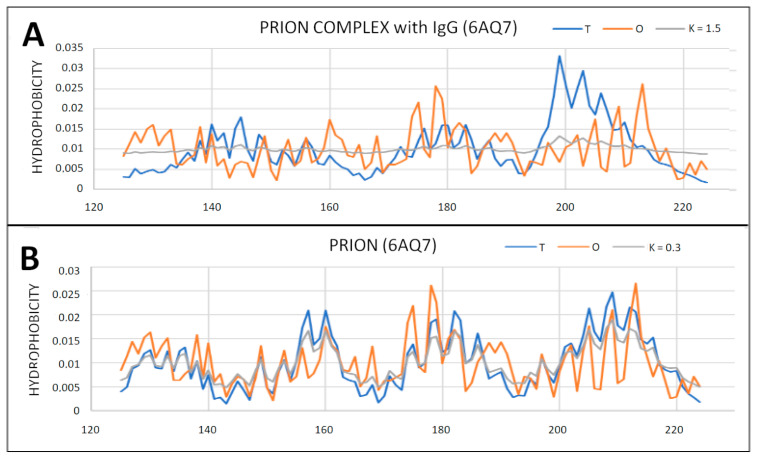
T, O and M profiles for prion protein available in PDB as 6AQ7 deposit. (**A**) Status of the prion protein as a component of the complex with the IgG Fab fragment (3D Gaussian function generated for the entire complex); (**B**) status of the prion protein treated as an individual structural unit (3D Gauss function generated independently for the prion protein).

**Figure 14:**
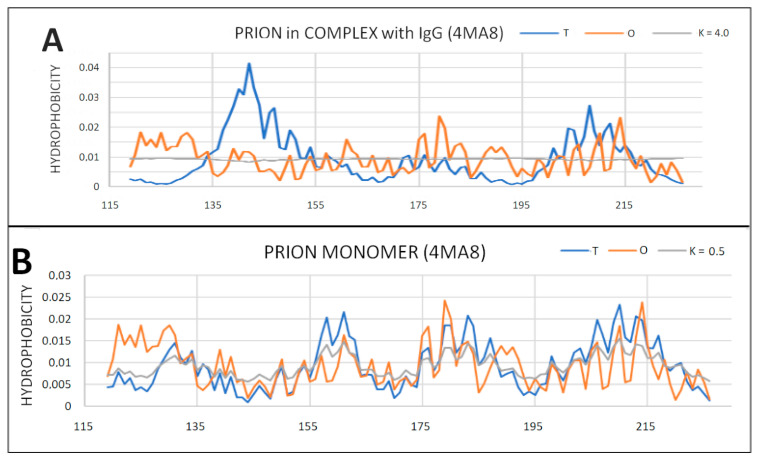
T, O and M profiles for the prion–protein complex with the Fab fragment of IgG. (**A**) Prion protein as part of the complex—3D Gaussian function generated for the complete complex; (**B**) prion protein treated as an individual structural unit (3D Gaussian function generated for prion protein individually).

Complexes of prion proteins with a Fab fragment of IgG are also available in the PDB database. This allows one to track changes resulting from interactions with other molecules. IgG Fab fragment molecules can be thought of as a target for the prion molecule. At the same time, it is possible to determine the influence of the presence of the Fab fragment of the IgG molecule on the structuring of the prion. With the very distant micelle-like status of the entire complex, the status of the individually treated prion proteins is not significantly altered. The changes in the parameters RD and K are observed depending on the composition of the interface. The protein itself maintains its close to micelle-like arrangement slightly above the RD threshold (RD > 0.5). Low values of the K parameter for prion proteins as components of the complex treated as individual structural units (3D Gauss function generated individually for the prion protein) indicate a relatively low degree of disturbance of the micelle-like system (Figure 12, Figure 13 and Figure 14).

The prion proteins treated as components of the complex show low adaptation to the structure of the complex treated as a form of micelles. High values of K parameter for prion proteins treated as components of the complex indicate the failure of these proteins to adapt to the expected structure of a common hydrophobic core in which the complexed molecule should participate. The monomer structure was changed by the presence of a target molecule in the case of 4MA8 where the value of RD exceeds 0.5 (Table 5 and Figure 14). The sections bounded by the disulfide bond retained the micelle-like status with respect to the prion as an individual unit.

#### 2.6.1. Amyloids

Amyloids are treated as highly specific structural systems creating linear fibrils with unlimited propagation. The structure of large-molecule complexes relies on the involvement of sidechain interaction in the stabilization of the complex. Amyloids represent a system that directly engages the backbone in interchain interactions [6]. Amyloids that appear as results of a PDB search using the keyword “amyloid” are given in Table 6. The summary available in this table includes amyloids consisting of a single fibril and superfibrils with different numbers of protofibrils (the number is given in Table 6 in parentheses).

The values distinguished as underlined (Table 6) distinguish examples of chains with higher RD status when threated as individual structural units. It is interpreted as better fitting to micelle-like organization in form of fibril. 

In light of the analysis based on the hydrophobicity distribution, amyloid 2KJ3 and 2RNM show a structure based on a centric hydrophobic core. Single chains in these fibrils show a status with parameter RD > 0.5. This means that the complex form is the preferred system form acceptable and supported by the aquatic environment. These two amyloids represent a system that adapts to the requirements and conditions of the aquatic environment. The only question is why single chains did not generate a globular structure with a hydrophobic core. Relatively low parameter values, comparable to those for globular proteins, suggest that the form of the complex in this case (2KJ3 and 2RNM) is entropically more favorable from the point of view of the reaction to the aquatic environment directing the structuring towards the centric hydrophobic core. 

**Figure 15:**
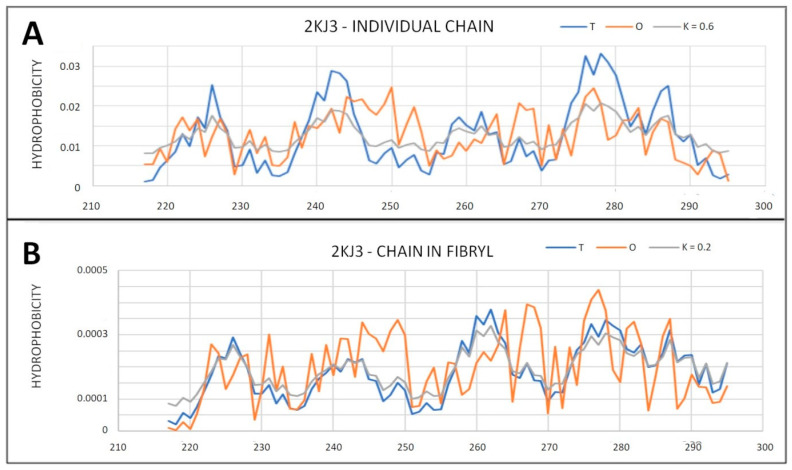
T, O and M profiles for amyloid showing a micelle-like structure—2KJ3. (**A**) Status of an individual chain; (**B**) Status of the chain as a component of the fibril.

**Figure 16:**
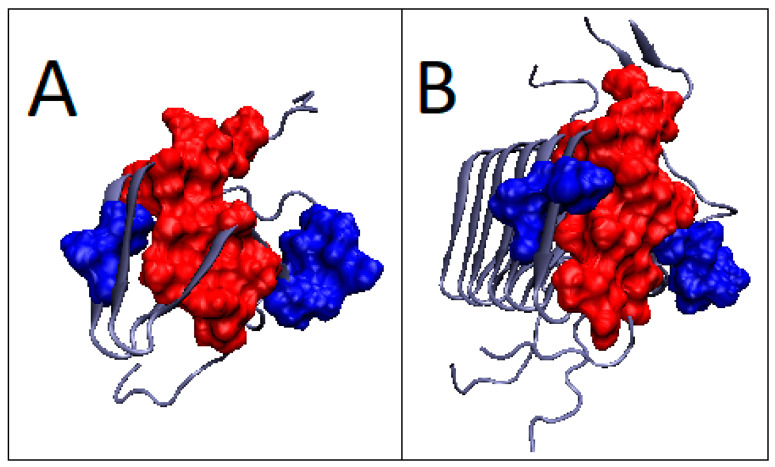
3D presentation of 2KJ3 amyloid structure showing micelle-like status. (**A**) 2KJ3—individual chain—red residues indicates components of the hydrophobic core determined on the basis of the profiles (from Figure 15A). (**B**) 2KJ3—chain as part of fibril—red residues indicate components of the hydrophobic core determined on the basis of the profiles (from Figure 15B). The residues distinguished as navy blue—the residues with a status inconsistent with the T distribution, determined on the basis of profiles in Figure 15.

Analysis of the T and O profiles for single chains in these amyloids shows local variations between T and O distributions (Figure 15 and Figure 16). Similarly, the 7LNA, despite the high RD value for the fibril, reveals the single chain status as being close to centric distribution. The values of K modify the T distribution to a slight extent (Figure 15B).

In the list of single-chain amyloids discussed here, 6EKA indicates a significant influence of an altered environment (K = 0.9) it is the degree identified for the membrane protein (rhodopsin [13]), i.e., the protein significantly influenced by a different environment, which is the cell membrane.

#### 2.6.2. Super-Fibrillary Amyloids

This group of prion amyloids is characterized by a very high values of RD and K parameters for both the proto-fibrils and super-fibrils form and for single chains.

**Figure 17:**
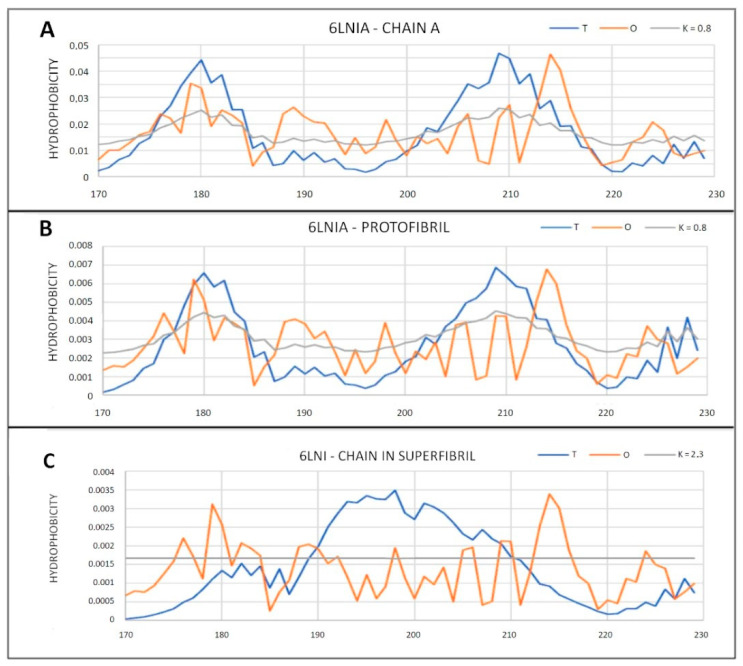
A set of profiles for amyloid showing a status significantly different from micelle-like with PDB ID 6LNI. (**A**) Individual chain; (**B**) status of chain in protofibril; (**C**) status in chain as part of super-fibril.

**Figure 18:**
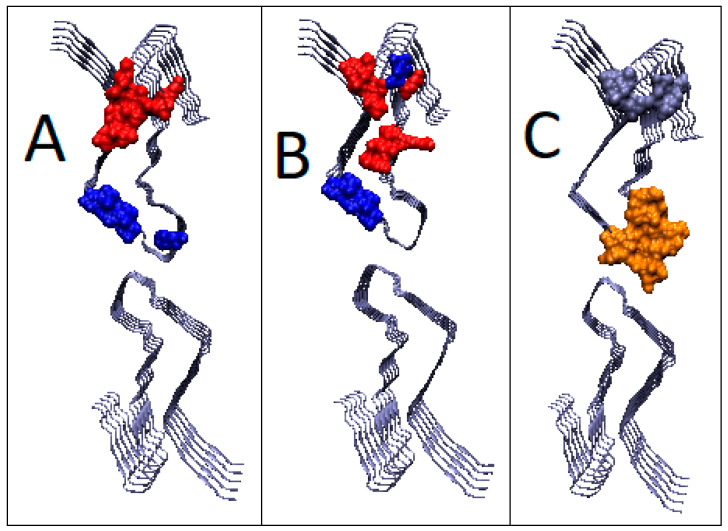
3D presentation of the structure of the super-fibril of the amyloid 6LNI. (**A**) Status of individual chain; (**B**) status as part of proto-fibril; (**C**) status of chain as part of super-fibril. Ice-blue—hydrophobicity excess; orange—hydrophobicity deficiency. Residues given in red—participating in hydrophobic core generation; residues in dark blue—residues representing different status in O profile than it is expected in T profile.

The presented T, O and M distributions for K = 2.3, identified to determine the status of the chain within the super-fibril, are interpreted as the effect of the structure’s independence from the environment. Obtaining distribution M visible in Figure 17 in the form of a straight line parallel to the *x*-axis means obtaining the status expressed by distribution R. It is a distribution with no differentiation in the levels of hydrophobicity depending on the location. The O distribution completely deviates from the T distribution (Figure 17C), representing a uniform system. The fluctuations that are present on the O profile are not, however, of any orderly character. Obtaining an O distribution of the R distribution type is treated as an expression of a separation from the environment. This state is interpreted as an analog of “vacuum.” The protein creates an environment for itself that cuts off both the aquatic environment (high K value) and the hydrophobic environment (straight line for the M distribution) (Figure 17C and Figure 18).

The status of the sections delimited by the Cys positions involved in the construction of the disulfide bonds is given in Table 7. The disulfide bond is present only in two examples.

**Figure 19:**
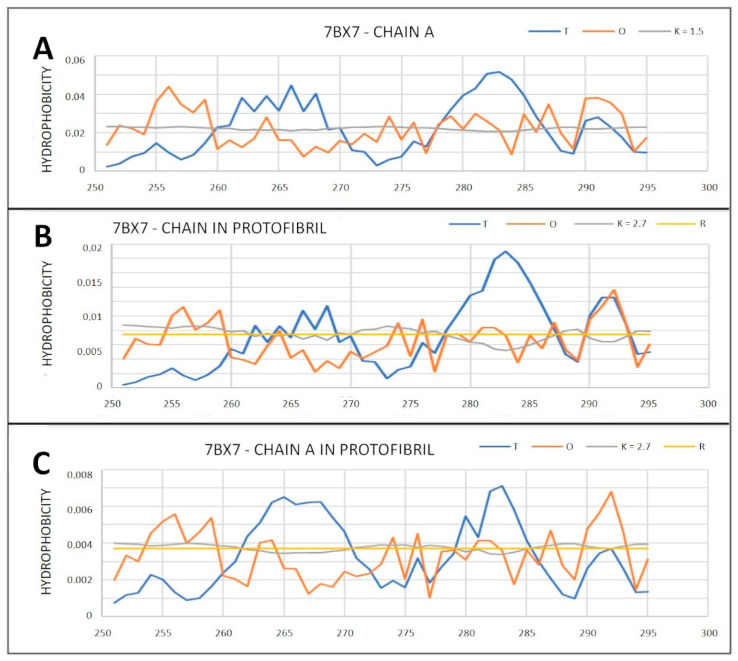
T, O and M profiles for determining amyloid status with PDB ID 7BX7. (**A**) Status of chain treated as an individual structural unit; (**B**) the status of the chain as a component of the proto-fibril; (**C**) the status of the chain as a component of a super-fibril.

The values shown in Table 7 suggest a significant deviation from the layout expected by the micelle-like one. This status is comparable to the status of elements with a disulfide bond (Table 6). An extreme example is represented by amyloid 7BX7 (Table 6). This applies to the status of complete super-fibril, protofibril and the individually treated polypeptide chain. This is illustrated by the set of profiles in Figure 19.

**Figure 20:**
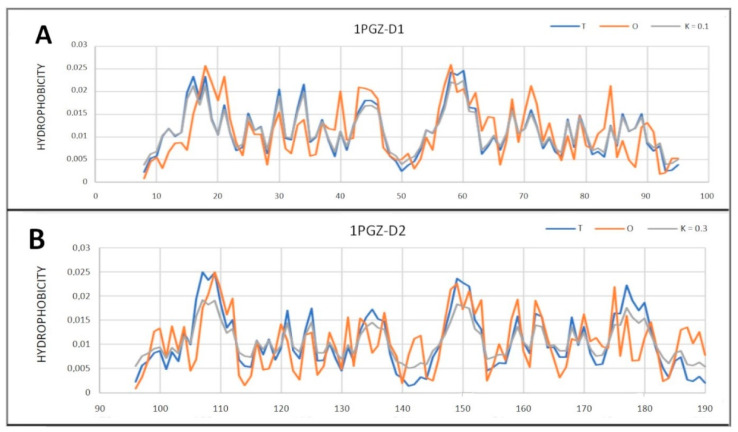
T, O and M profiles for the reference protein (in relation to 7BX7) of the hnrnpa family with PDB ID 1PGZ. (**A**) Domain 1; (**B**) domain 2.

The amyloid in question belongs to the heterogeneous nuclear ribonucleoprotein A1 from the Homo sapiens (hnrnpa) family. Despite the lack of available native chain structure with identical sequence, an analysis of a representative of this family (1PGZ) was performed. It turns out that each of the two domains that make up this protein represents a micelle-like system (Figure 20). Thus, this is another example of a radical change in the hydrophobicity distribution in the transition from the native form to the amyloid form.

## 3. Discussion

The proposed model of the external force field created by the water environment has the character of the continuous field in contrast to the models treating water as a set of single molecules [6]. Moreover, the proposed model identifies the properties of the environment on the basis of its effect on the polypeptide chain. The FOD model uses a 3D Gauss function to express the effect inducing the formation of a centric hydrophobic core. The FOD-M model introduces modifications resulting from the need to take into account the hydrophobic environment for proteins anchored in the membrane. Initially it was assumed that the membrane proteins represent the degradation of T_MAX_-Ti. However, it turned out that the presence of a 3D Gauss (Ti) component is needed. Modification of the 3D Gauss function with a different degree of this modification (parameter K) turned out to express the influence of other factors, not necessarily strictly hydrophobic. The transformation from the native to the amyloid form achieved with the gradually increasing concentration of tri-fluoro-ethanol is the result of a fundamental change in water structuring characteristics [56].

The form and strength of the external 3D Gaussian field interaction for globular proteins has been modified to the 2D Gauss form [57] favoring the orientation of the polypeptide chain structuring to the flat form, which is present in all known amyloids. Similarly, F19S and G37D mutations introduced into Aβ (1-42) causing resistance to amyloid transformation are due to a significant change in the hydrophobicity of the altered residues. This probably results in the possibility of directing folding towards the generation of a centric hydrophobic core [58]. The evidence may be a set of proteins that differ by a single mutation, which results in a fundamental change in structuring (helices replaced with Beta-structural fragments) [59,60]. If such a fundamental change in the secondary structure system is possible under the influence of a single mutation, then a significant change in the Aβ (1-42) chain, introduced in the discussed mutation, may result in the preference or exclusion of the amyloid transformation.

In the FOD-M model used, the role of the so-called rack—a permanent chaperon—that imposes structuring becomes visible. The matching structure to the target appears to be in proteins stabilized by the presence of a hydrophobic core. It should be noted that the structure with a well-defined hydrophobic core (RD low and K = 0) turns out to be stable under the condition of an appropriate environment, i.e., standard conditions. If this environment changes, e.g., due to the presence of a target molecule, the form of the external environmental force field changes. Proteins with K = 0 owe their structure to the force field generated by water. Each change within this field is a factor that determines protein structuring and affects the structural form of a given protein [61,62].

The fuzzy oil drop model, especially its modified form, taking into account the presence of other factors seems to reflect the phenomena of structural variability, which results from the continuous form of this model. It is difficult for discrete models to deal with this phenomenon [63]. Amyloid transformation is often associated with intrinsically disordered proteins [64,65]. The analysis of this group of proteins is planned in the near future.

## 4. Materials and Methods

### 4.1. Programs Used

**Figure 21:**
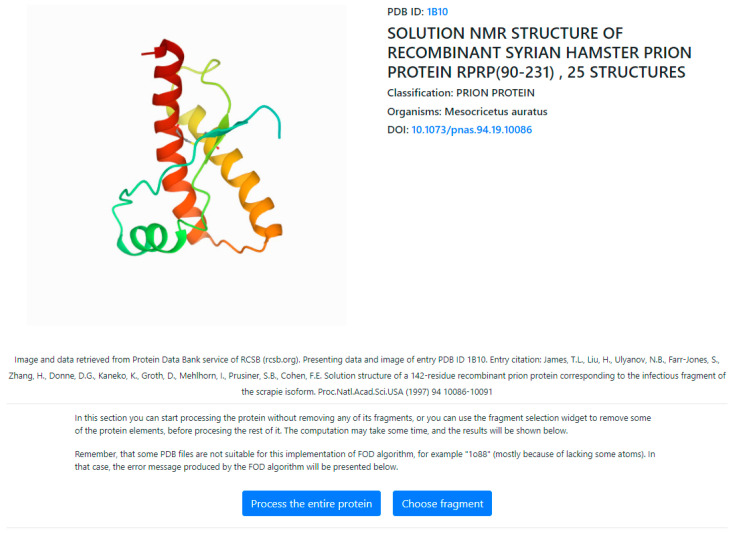
User interface of the HPHOB online tool that provides the ability to compute FOD model parameters for arbitrary protein structures. The tool is available at https://hphob.sano.science.

The potential used has two possible access to the program:

1. The program allowing calculation of RD is accessible upon request on CodeOcean platform: [66] https://codeocean.com/capsule/3084411/tree. Please contact the corresponding author to obtain access to your private program instance.
2. In order to ensure reproducibility of results and provide easy access to the computations discussed in this paper, the authors have also implemented an online tool where the FOD computations can be performed on arbitrary protein structures, including the structures discussed in this paper. The application—implemented in collaboration with the Sano Centre for Computational Medicine (https://sano.science, accessed on November 2021) and running on resources contributed by ACC Cyfronet AGH (https://www.cyfronet.pl, accessed on November 2021) in the framework of the PL-Grid Infrastructure (https://plgrid.pl, accessed on December 2021)—provides a web wrapper for the abovementioned computational component, and it is freely available at [67] https://hphob.sano.science—the dialog window shown in Figure 21.

The tool enables users to select a protein structure by entering its PDB identifier, then allows the selection of specific parts of the protein (including individual chains and secondary folds—all the way down to individual residues) and finally to run the FOD computation on the selected fragments in order to obtain RD and hydrophobicity distribution data (Figure 21).

The program used to present the 3D structures of proteins is VMD [68]. https://www.ks.uiuc.edu/Research/vmd/ (accessed on 21 November 2021) [69].

### 4.2. Data

The proteins analyzed in the present study are summarized in Table S1—Supplementary Materials.

## 5. Conclusions

Two premises have been included in the current analysis of prion proteins and their amyloid forms:

1. Fundamental change in the strategy of creating large-molecular structures by amyloids in relation to protein complexes;
2. Participation of the environment in the discussed process, resulting in the conclusion that the modification of the environment may result in amyloid transformation of almost any protein [56].

Complexes are formed on the basis of interactions between the side chains, unlike amyloids, for which its structure of fibrillary forms results from the involvement of the backbone itself. This phenomenon is interpreted by the FOD model as a change in the participation of the environment from 3D Gauss to 2D Gauss [57], which is obvious when taking into account the flat structure of the chains included in all amyloids available so far. This change of environment reflects the mechanism of obtaining amyloid structures by shaking, which evidently introduces 2D structuring as a result of the increasing proportion of interphase structuring of water. A similar change may arise in the presence of other environmental factors that alter the structure of the water. The natural environment is known as “overcrowded” [70,71].

Therefore, sensitivity to the presence of numerous factors must be encoded in the structure of proteins. The applied FOD-M model responds to these conditions. 

Two scenarios for the amyloid transformation have been proposed in [13]. One of them is expressed by a change in the protein status from the form with a high value of K parameter for the native form and a low value of K for the amyloid form. This scenario concerns A-synuclein, which under normal conditions requires a rack to stabilize its biologically active structure—also known as a permanent chaperone. Thus, the influence of the environment is significant. The release of this protein from the influence of the permanent chaperone results in a natural adaptation process to the conditions of the aquatic environment; therefore, A-synuclein amyloid takes the form of a micelle-like with a low value of K. The reverse pattern applies to the amyloid composed of the chains of the IgG V domain, which under natural conditions is very close to micelle-like, while the amyloid exhibits high values of K, which is interpreted as a significant contribution of the environment to the stabilization of the system with high values of K. The amyloid structure of IgG-derived light-chain discussed here does not exhaust the topic. In fact, there are known observations on the amyloidogenic properties of the IgG heavy chain as well [72].

The present analysis is a continuation of the research for the given interpretation [13]. Supplementing it with an analysis of intrinsically disordered proteins will allow for a wider speculation about the mechanism of amyloid transformation (in preparation). 

Interpretation of K values allows assessment of the external field participating in the fibril/superfibril construction. In particular, the positions (distinguished as bold) with K > 1.0 reveal the role of the environment. Positions with RD > 2.0 suggest the support from other external factors. The water solution seems not to be the power keeping these constructions stable. If the native form of these proteins is known, a mutual comparison can make possible the recognition of the mechanism behind the transformation, as it was shown in [13]. In contrast to 2KJ3 and 2RNM, the fibrillar form seems to be an effect of an adaptation to the water environment since the micelle-like distribution (preferable by water environment) is generated. The interpretation given in [13] can be applied here. 

Summarizing the presented analysis, it should be stressed that the applied model seems to identify the contribution of external factors influencing the structuring of amyloid forms. We can distinguish (Table 6) fibrils suggesting the participation of aqueous environment resulting in the formation of a form characteristic for micelle-like structure with centrally located hydrophobic cores. Next to them, there were identified forms characterized by high RD and K values, which, as it was shown by fuzzy oil drop model, require participation of other (in addition to water) factors actively influencing amyloid structuring. Experimental studies provide information on this subject. They are identified, for example, in Parkinson’s disease (PD); aSyn is known to trans-synaptically spread from neuron-to-neuron recruiting endogenous monomers in the infected cells into a pathological conformational prion-like templating process [72,73]. In addition, aSyn’s interaction with non-amyloidogenic intracellular protein partners redirects its pathological polymerization into different structural polymorphs [74,75]. Taken together, the existence of multiple “strains” with different ultrastructural and biological features might explain the clinical heterogeneity among PD and related a-Synucleinopathies. 

The introduced K parameter in the FOD-M model seems to assess the strength with which the environment of a given protein influences structuration, since alteration brings micelle-like forms (centric hydrophobic core) to forms far from those typical for the aqueous environment. The contribution of the “rack” to the maintenance of the biologically active structure (its influence is determined by the K parameter) is demonstrated by experimental studies.

## Figures and Tables

**Table 1:** The values of the parameters RD and K for the considered single-chain prion proteins.

PDB-ID	Chain		Fragment Limited by SS-Bond	
	RD	K	RD	K
1HJN	0.232	0.0	0.255	0.1
1HJM	0.373	0.2	0.376	0.1
6FNV	0.385	0.0	0.469	0.0
1E1W	0.388	0.2	0.352	0.1
1DX0	0.412	0.2	0.393	0.0
6DU9	0.425	0.3	0.413	0.2
1QM3	0.441	0.3	0.413	0.2
1FKC	0.448	0.3	0.470	0.3
1DX1	0.452	0.3	0.396	0.1
1AG2	0.454	0.3	0.455	0.3
1DWZ	0.457	0.3	0.394	0.1
1QLZ	0.471	0.3	0.472	0.2

**Table 2:** Summary of the values of parameters RD and K describing the status of partially unfolded prion proteins (the values for the indicated sections are given).

Protein	Chain		Fragment Limited by SS-Bond	
	RD	K	RD	K
5YJ5	0.539	0.5	0.466	0.0
90–145	0.751	2.3		
145–231	0.513	0.4	0.524	0.3
6FNV	0.583	0.5	0.536	0.3
90–145	0.765	3.2		
145–233	0.491	0.4	0.506	0.3
5L6R	0.622	0.5	0.456	0.0
90–130	0.776	2.1		
130–234	0.512	0.3	0.547	0.3
5YJ4	0.639	0.5	0.372	0.4
90–140	0.712	2.1		
140–231	0.416	0.2	0.352	0.1

**Table 3:** Prion proteins for which their structure available in PDB are dimers.

PDB-ID	Dimer/Monomer		Status as Part of Complex		Fragment Limited by SS-Bond	
	RD	K	RD	K	RD	K
4HLS—AB	0.547	0.4				
A	0.413	0.3	0.536	0.5	0.445	0.2
B	0.440	0.3	0.555	0.4	0.361	0.1
4HMM—AB	0.544	0.4				
A	0.418	0.3	0.543	0.5	0.449	0.25
B	0.428	0.3	0.543	0.4	0.357	0.1
4HMR—AB	0.530	0.4				
A	0.434	0.3	0.520	0.4	0.451	0.2
B	0.429	0.3	0.539	0.4	0.360	0.1

**Table 4:** The parameters describing the status of prion-like proteins.

PDB-ID	Chain		Fragment Limited by SS-Bond	
	RD	K	RD	K
1LG4	0.484	0.4	0.494	0.3
1I17	0.427	0.2	0.373	0.1

**Table 5:** Prion molecules in complex with fragment Fab of IgG–PDB-ID, with P added to the PDB ID denoting prion chain treated as an individual chain.

PDB-ID	Complex/Monomer		Status as Part of Complex		Fragment Limited by SS-Bond	
	RD	K	RD	K	RD	K
6AQ7	0.717					
6AQ7-P	0.466	0.3	0.655	1.5	0.438	0.2
4YXL	0.752					
4YXL-P	0.500	0.4	0.761	2.7	0.438	0.2
4MA7	0.759					
4MA7-P	0.481	0.4	0.766	2.4	0.434	0.2
4MA8	0.779					
4MA8-P	0.525	0.5	0.812	4.0	0.447	0.2

**Table 6:** List of amyloid proteins appearing in the PDB database after the use of the keyword “amyloid.” A set of parameters was provided for both the proto-fibril and super-fibril forms as well as for the single chain and the common floor (in the case of a super-fibril). The number of proto-fibrils included in the super-fibril is given in brackets. The 1PGZ item is a protein in globular form belonging to the hnrnpa family added to the amyloid pool to allow for comparative analysis against 7BX7. The values in bold—the highest discordance versus micelle-like hydrophobicity distribution. The underlined positions—examples with individual chain of higher RD status with respect to super-fibril.

PDB-ID	Super-Fibril		Proto-Fibril		One Level		Chain—Individual	
	RD	K	RD	K	RD	K	RD	K
2KJ3	0.418	0.2					0.618	0.6
2RNM	0.446	0.3					0.644	0.8
2MUS	0.596	0.6					0.636	0.6
2LBU	0.604	0.6					0.652	0.8
7LNA	0.612	0.6					0.568	0.5
5W3N	0.641	0.6					0.749	0.7
6EKA	0.666	0.9					0.660	0.7
6UUR	0.668	0.7	**0.745**	**1.0**	0.659	0.6	0.730	0.8
**6ZCF (2)**	**0.716**	**1.5**	0.620	0.8	**0.756**	**1.6**	**0.695**	**1.0**
**6ZCG (4)**	**0.778**	**2.0**	0.623	0.9	**0.828**	**2.1**	0.673	0.9
**6LNI (2)**	**0.794**	**2.3**	0.624	0.8	**0.810**	**2.2**	0.663	0.8
**6VPS (3)**	**0.814**	**1.6**	**0.790**	**1.3**	**0.827**	**1.1**	**0.806**	**1.1**
**7BX7 (2)**	**0.823**	**2.7**	**0.790**	**2.7**	**0.831**	**2.7**	**0.789**	**1.5**
1PGZ	0.582	0.6						
D1 (8–95)	0.319	0.1						
D2 (96–190)	0.454	0.3						

**Table 7:** Status of fragments delimited by SS-bonds.

PDB-ID	Fibril Superfibril		Proto-Fibril		Chain—Individual	
	RD	K	RD	K	RD	K
7LNA	0.718	1.0			0.706	1
6LNI (2)	0.717	2.2	0.650	0.7	0.708	0.7

## Data Availability

All data can be available upon request addressed to corresponding author. The program allowing calculation of RD is accessible on GitHub platform: https://github.com/KatarzynaStapor/FODmodel and on platform (accessed on 21 November 2021) and https://hphob.sano.science (accessed on 21 November 2021).

## Supplementary Materials

The following are available online at https://www.mdpi.com/article/10.3390/ijms222413494/s1.
