# Supplementary material for: On the Dependence of Prion and Amyloid Structure on the Folding Environment
Source: Int J Mol Sci. 2021 Dec 16;22(24):13494. doi: 10.3390/ijms222413494 (PMC8707753; doi:10.3390/ijms222413494)
Supplement: Supplementary file 1 [file ijms-22-13494-s001.zip › ijms-1494783-supplementary.pdf]

Supplementary Material

On the Dependence of Prion and Amyloid Structure on the Folding Environment

Irena Roterman *, Katarzyna Stapor, Krzysztof Gądek,Tomasz Gubała, Piotr Nowakowski, Piotr Fabian and Leszek Konieczny

Table S1. List of proteins analysed in the paper. The numbers in parentheses in column PDB ID–number of protofibrils in superfibrils.

PDB-ID		One Chain Prion Proteins	
1HJN	Homo sapiens	Major prion protein precursor	[25]
1HJM	Homo sapiens	prion protein at pH 7.0	[25]
1E1W	Homo sapiens	prion protein variantRr220K	[26]
1DX0	Bos taurus	prion protein residues 23-230	[27]
6DU9	Homo sapiens	Major prion protein	[28]
1QM3	Homo sapiens	prion protein fragment 121-230	[29]
1QLZ	Homo sapiens	Major prion protein	[29]
1FKC	Homo sapiens	Major prion mutant E200K	[30]
1DX1	Bos taurus	prion protein residues 23-230	[31]
1DWZ	Bos taurus	Major prion protein	[31]
1AG2	Mus musculus	Major prion protein	[32]
		**Partially Unforded Prion Proteins**	
5YJ5	Homo sapiens	wildtype human prion protein (m129)	[33]
5YJ4	Homo sapiens	mutant g127v of human prion protein	[33]
6FNV	Odocoileus hemionus	Major prion protein	[34]
5L6R	Homo sapiens	truncated human prion protein	[35]
		**Prion Proteins in Form of Dimers**	
4HLS	Oryctolagus cuniculu	Mutant Major prion protein	[36]
4HMM	Oryctolagus cuniculus	Major prion protein	[36]
4HMR	Oryctolagus cuniculus	Major prion protein	[36]
		**Prion-Like Proteins**	
1LG4	Homo sapiens	human doppel protein fragment 24-152	[37]
1I17	Mus musculus	mouse doppel 51-157	[38]
		**Prion Proteins in Complex with Fab Fragment of IgG**	
6AQ7	Mus musculus	Major prion protein	[39]
4YXL	Mesocricetus auratus	Major prion protein from	[40]
4MA7	Mus musculus	unp residues 116-229	[41]
4MA8	Mus musculus	Major prion protein	[41]
		**Prion Proteins in Form of Amyloids**	
2KJ3	Podospora anserin	het-s(218-289)	[42]
2RNM	Podospora anserina	het-s(218-289)	[43]
2MUS	Podospora anserinA	(PrP(C))	[44]
2LBU	Podospora anserinA	Small s protein	[45]
7LNA	Mesocricetus auratus	prp27-30, prp33-35c	[46]
5W3N	Homo sapiens	RNA-binding protein fus	[47]
6EKA	Podospora anserina	DNA chromosome 3	[48]
6UUR	Homo sapiens	Human prion protein fibril, m129 variant	[49]
6ZCF (2)	Mus musculus	Serum amyloid a-2 protein	[50]
6ZCG (4)	Mus musculus	murine saa1	[50]
6LNI (2)	Homo sapiens	full-length human prion protein	[51]

6VPS (3)	Drosophila melanogaster	Translational regulator orb2	[52]
7BX7 (2)	Homo sapiens	Heterogeneous nuclear ribonucleoprotein a1	[53]
	**Reference Protein hnrnpa for Comparable Analysis with 7BX7**		
1PGZ	synthetic	telomeric repeat d(ttaggg)n	[54]
